# Fish faddism causing low-level mercury poisoning in the Caribbean: two case reports

**DOI:** 10.1186/1757-1626-0002-0000007009

**Published:** 2009-04-29

**Authors:** Lexley M Pinto Pereira, Surujpaul Teelucksingh

**Affiliations:** 1 Departments of Para-clinical Sciences and Medicine, Faculty of Medical Sciences, The University of the West Indies, St Augustine, Trinidad and Tobago, West Indies

## Abstract

Two otherwise healthy middle-aged males presented with persistent abdominal and lower- back pain, progressive weakness, paraesthesias, fatigue and weight loss over 8-12 months. Extensive work-up failed to localize organ pathology. Both men, strongly aware of the nutritional benefits of fish had a diet dedicated of canned and fresh fish. Raised blood mercury levels confirmed clinical suspicion and serial levels declined with symptom resolution after excluding dietary fish. To gain reported health benefits of fish as a healthy food modest consumption is encouraged. Efforts to monitor fish consumption and mercury residues in fish are recommended in Trinidad and Tobago.

## Introduction

As an excellent source of protein with low saturated fat content fish is a healthy food choice. Yet it presents significant environmental health interest because mercury in freshwater and marine food chains presents an avenue for human exposure. Humans are exposed to methylmercury, the organic form present in trace amounts in nearly all sea food and fish and shell fish contain the highest amount of mercury in the human diet [1]. Predatory fish have higher levels than non-predatory fish at the lower rank of the food chain. Methylmercury is absorbed from water passing over the gills, is tightly bound to fish tissue proteins, has an exceptionally long biological half-life, is scarcely reduced by cooking and comprises approximately 85% of mercury in fish which is reflected in whole blood mercury concentrations [2]. Consequently mercury levels in blood depend on the species and the quantity of fish consumed [3]. The Minamata disease of 1956 and the second outbreak in 1965 of Minamata Niigata disease, from consuming fish with mercury burdens ranging from 9 to 24 μgm g(-1) clearly illustrate the dangers from consuming large amounts of methylmercury accumulated in seafood [4]. Toxicological consequences of chronic low-level mercury exposure from habitual seafood consumption remain an area of concern.

## Case presentations

We report on mercury exposure in two patients who consumed a dedicated fish diet in an extremist pursuit of health zeal. Both patients were non-smokers, social drinkers, without any particular contributory family history and were otherwise well.

### Case report 1

Patient 1 was a 38-year-old male marine mechanic of mixed Caucasian and African ancestry. He had mild pain and swelling in his left testicle, which he attributed to a sports injury. Subsequently he suffered generalized weakness, diarrhea, fever, lower back pain and abdominal pain. Urinalysis, semen analysis, kidney, prostate and bladder ultrasound were normal and he was put on co-amoxiclav for epididymitis. His worsening symptoms were partially relieved by ibuprofen. He told of a 'stomach gas' pain which was aggravated by co-amoxiclav, but decreased the epididymal swelling. Weakness persisted and was accompanied by sore throat, pains in the hip, eyes and ears. Complete blood count, electrolytes, tests for thyroid, liver and kidney function, were normal. He intermittently received antibacterials for prostatitis.

Due to the chronic and severe abdominal 'gas' pains a colonoscopy was performed and some relief came from mesalamine prescribed for colitis. A nodal swelling under his chin evaluated by MRI and screening for an autoimmune disease were non-contributory. Weight loss of 10 lbs in five months was accompanied by persistent weakness with lack of energy primarily so that he could work for just half a day. His mother said he forgot routine household tasks and found it difficult to extract himself out of bed. Believing he may have been exposed to heavy metal poisoning from paint solvents at work, the patient voluntarily tested for blood levels of lead and mercury. Lead was not elevated; whole blood mercury was 17.8 ug/L.

On questioning he admitted to sleep disturbances, depressive episodes, pins and needles sensations, discolored stools, muscle pains, leg cramps, joint pains, back ache of severe intensity, with intense fatigue. There were no contributory physical signs. As he was extremely health conscious the nature of his diet was explored. When rumours of 'bird-flu' hit Trinidad, he drastically changed his diet from chicken to fish alone, basically tuna and shark, for all meals for approximately 6 months. When he was seen he had already been examined at the Cleveland clinic and advised treatment with penicillamine. He voluntarily tested for mercury 3 months later at which time, the level was 13 ugm/L. When the authors saw him, he was advised to stop all fish and retest for mercury after the next three months to observe any decline based on a two month half-life; treatment with dimercaptosuccinic acid (DMSA) was available if indicated. Whole blood mercury levels on three subsequent occasions at intervals of 3 months were 5.6 ugm/L, 2.3 ugm/L and <2 ugm/L. At the time of the last test his pains had practically disappeared, he demonstrated weight gain, had an outgoing disposition and resumed normal working hours.

### Case report 2

The second patient was a 46 year male chief executive officer of mixed Asian and Caucasian heritage, who reported a 'fluttering' in the chest. Holter monitoring, stress test and echocardiogram were normal. He felt 'washed out'. Subsequently he developed a dull aching discomfort on the left side of the abdomen typically occurring when eating and about 2 hours after eating. The pain woke him at night lasted 1-1.5 hours and was not related to any specific foods, but seemed to worsen when he was hungry. Based on a past history of heartburn he received omeprazole, which offered some benefit but the nocturnal awakenings continued. He developed lower pelvic discomfort radiating from the hips to both legs. He had persistent nausea, soft stools and lost approximately 16 pounds over a year; he was unable to do his routine exercise. Abdominal CT scan and MRI revealed a renal cyst, gastro-intestinal endoscopies and biopsies were normal. Extensive investigations for *H pylori*, hepatitis A and C, amylase, lipase, ANCA for IBD, tissue transglutaminase for coeliac disease, ANF and anti-ds DNA were negative. Now his major concern was pain, weakness and numbness in the lower back, hips and thighs and with continuing generalized weakness and malaise. He was evaluated at the Mayo clinic at Jacksonville and treated for degenerative disk disease.

On questioning, he admitted to having a dedicated diet of fish, mainly canned tuna and fresh shark for health reasons. Mercury evaluated in whole blood was 26.3 ugm/L. After ceasing fish consumption blood mercury evaluations at just over two monthly intervals were 19.9, 14.4, 11.3, 7.1 and 3.3 ugm/L. About 16 months after his initial complaints, muscle weakness and abdominal discomfort had disappeared; his energy level was had returned and he enjoyed his work.

## Discussion

Both patients reported primarily gastro-intestinal (vague abdominal discomfort difficult to localize), CNS (extreme fatigue, paraesthesias and sleep disturbances) and integumetary (muscle pains, joint pains) complaints with deterioration in general status (weight and energy loss). The persistence of both patients, that 'something was wrong' despite all investigations returning negative was notable. Mercury exposure is insidious and presents in a nebulous fashion. The primary source of human exposure to environmental mercury is through seafood consumption. Bioaccumulation of mercury occurs in aquatic environments, from long-lived piscivorous fishes and marine mammals who have a mercury burden one-million times that of the surrounding water body, typically exceeding 1 μgm(-1). Mercury bioaccumulates up the food chain and large predatory species like tuna, shark and swordfish have high concentrations of mercury in their tissues [5]. Fish-associated mercury exposure ranging from approximately 30μgm/L to >140μgm/L has been reported in the United States [6]. In San Francisco, Hightower and Moore identified seriously high elevated whole blood mercury (up to 90μgm/L) in a series of affluent patients, raising concerns that upper scale fish consumers may be ingesting enough methylmercury to cause clinical illness [7]. Toxicity of organic mercury exposure may be delayed from weeks to months with predominant gastro-intestinal and central nervous system effects [8]. Sensory peripheral neuropathy with low to moderate chronic exposure is common, and central nervous system effects include personality changes, irritability, fatigue, tremor (usually intention tremor), ataxia, difficulties with memory and concentration, sleep disturbances and a metallic taste [9].

Occupational hazards, with high suspicion index of dietary changes and symptoms may clue into the possibility of exposure. Diagnosis was elusive in the second patient and was by omission after extensive investigation at home and abroad. His paraesthesias (usually the first symptoms) attributed to disk injury, emphasize the condition can be missed unless suspected.

Identifying and removing the source of mercury is crucial. Even though the levels were not remarkably high, after about three months of a fish free diet both patients felt near normal after approximately 12 months from the time they were first seen and advised to discontinue fish due to suspected mercury exposure. With a half-life of 70 days, at least five half-lives would need to be completed for the steady state levels of organic mercury to drop and with a return of a sense of comparative well being. Mercury toxicity is partially or wholly reversible through natural elimination. Both patients did not need chelation therapy as blood mercury was well below 40μgm/L and reducing the source of exposure was sufficient to decrease the body burden of mercury [8]. They were encouraged to return fish gradually to their nutritional regime in modest amounts and to avoid consistently excess fish intake. Fish consumption being promoted as preventing heart disease and good nutrition, it is important to monitor for individuals displaying the effects of excess intake, particularly in island nations where fish is available year-round. To our knowledge these are the first reports of excess fish consumption causing organic mercury poisoning, in the Caribbean where fish is a daily component of the islanders' diet. In Trinidad and Tobago shark is a 'staple' fish, 'shark and bake' is an extremely popular fast food and curried shark is a favorite meal. Considering the easy availability of fish, the Ministry of Health should make advisories available on avoiding fish excesses and the risk of mercury excess and accumulation during a lifetime of fish consumption. Such advice would be important particularly for the enthusiasts of 'shark and bake' in which the meat comes from huge shark as opposed to the domestic prepared meals made from smaller animals.

Nearly all fish contain some amounts of methyl mercury, including canned tuna which though containing lower levels of mercury is a cause for concern in a dedicated diet. Canadian advisories recommend one meal (150 gms) per week of shark, swordfish and fresh and frozen tuna, with no more than a meal per month in children and pregnant women [10]. Toxicologists at the United States FDA advise consumption of fish with high mercury content such as shark and swordfish be limited to one serving (7 ounces) per week for persons other than pregnant women and women of childbearing age who may become pregnant, (a serving per month) [11]. Fish safety experts note some cans have higher than average mercury content, posing uncertainty on their safety particularly for feto-maternal transfer. The mean level of mercury in canned white tuna has been shown to be significantly higher than that used by the United States FDA in its risk assessment and public information, calling for systematic monitoring of canned fish supply for mercury content. Awareness about mercury toxicity particularly in white canned tuna, has adversely affected the United States canned tuna industry [12].

Pisciculture is an industry in the Caribbean, as in many islands and coastal areas and catches of fish, include shark, kingfish, grouper, redfish, snapper, shrimp, and tuna. As far as these authors are aware there are no data available on mercury residues in local fish. Efforts to monitor fish consumption and mercury residues in fish to study the potential toxicity of methylmercury exposure in individuals consuming large amounts of fish with mercury residues are recommended. Several locals are involved in the fish industry and with its easy availability on the islands fish is a popular food component These islands offer a fertile site to search for more information to recognize the effects of long-term exposure to low levels of methylmercury. After all toxicology lives by the maxim, 'the dose makes the poison.

## Conclusions

In both cases, the causative association of high mercury levels with the symptoms was made admittedly by a process of omission. The objectivity was provided by the levels of blood mercury, which showed a decline following cessation of the presumed source of mercury contamination. Fish as a 'good' dietary component can turn noxious in excess.

### Patient's perspective (Case 2)

I am health conscious, eat a balanced diet, exercise regularly and have detailed regular medical examinations. In May 2006 I started to feel very drained and experienced a strange fluttering in my chest on exercising, a burning sensation and persistent dull ache in my stomach region. Nexium (esomeprazole) prescribed for suggested gastric acid reflux, made me worse. My energy level fell, sleep was disturbed and I was generally worried. An upper gastroenterology endoscopy and biopsy confirmed no abnormalities. An ECG, stress test, echo cardiogram, 24 hour Holter monitoring abdominal CT and MRI scans were clean. The dull stomach pain migrated to my chest, sleeping disturbances worsened, panic and anxiety attacks came on and my energy level deteriorated. On exercising I felt nauseous and drained. The stomach burning, sleep disturbances, dull chest aches and occasional chest flutter were unrelenting. Another gastroenterology referral, more tests, an upper endoscopy and colonoscopy, were inconclusive except for a high antibody Epsom Barr virus titre. The diagnosis was either a post viral syndrome, acid reflux and/or irritable bowel syndrome. Pariet (rabeprazole) and Motilium (domperidome) were prescribed; my symptoms persisted. Now an anxiety disorder was diagnosed.

Early in 2007 I stopped the acid control medication which seemed to make me worse. The dull ache in the stomach went to the lower abdomen. Nausea, frequent anxiety attacks, disturbed sleep and very low energy level continued; lower back discomfort now came on. Investigations for heavy metals, allergies and Lupus revealed high blood mercury of 26 μgms/L (N=5μgms/L). The explanation lay in my 2-3 year self imposed dietary modification of fish contributing my protein; it was fish at every meal, lots of tuna, king mackerel and occasionally groupa. Fish was eliminated from my diet and serial blood mercury levels were monitored. In July 2007 with intense pain in the back, hips and legs, generalized severe weakness, and feeling an all time physically low I approached The Mayo Clinic at Jacksonville (Florida) to review my case. After extensive blood tests, abdominal CT scan, MRI of lumbar and thoracic spine the possible diagnosis was irritable bowel syndrome. The confirmed high mercury level was not felt to be causative.

Early in 2008 (still on a diet without fish) I gradually improved and by the year-end had only minor stomach, leg and lower back discomfort. I resumed exercising, generally felt much better and able to cope. At the last test in August 2008 blood mercury level was 3.5μgms/L; and fish was still out of my diet. Having read extensively on mercury I cannot confidently conclude my problems resulted from high mercury levels; but there is no other explanation. The Hightower Report (http://diagnosismercury.org/) without appropriate controls has been criticized. I am now very careful about what I eat, am particularly attentive to possible contaminants and approach all things in moderation.

## List of abbreviations

MRI: Magnetic resonance imaging; DMSA: Dimercaptosuccinic acid; ANCA: Anti-neutrophil cytoplasmic antibodies; IBD: Inflammatory bowel disease; ANF: Anti-nuclear antibody; CNS: Central Nervous System; FDA: Food and Drug Administration; CT: Computer Tomography; DNA: Deoxyribonucleic acid.

## Consent

Written informed consent was obtained from the patients for publication of this case report. A copy of the written consent is available for review by the Editor-in-Chief of this journal.

## Competing interests

The authors declare that they have no competing interests.

## Authors' contributions

Both LPP and ST acquired the data, reviewed and evaluated the patients' data regarding blood levels and toxicity. LPP was a major contributor in writing the manuscript and ST critiqued it. LPP and ST read and approved the final manuscript.
